# Impaired Expression of Humanin during Adrenocortical Carcinoma

**DOI:** 10.3390/ijms25021038

**Published:** 2024-01-15

**Authors:** Małgorzata Blatkiewicz, Marta Szyszka, Anna Olechnowicz, Kacper Kamiński, Karol Jopek, Hanna Komarowska, Marianna Tyczewska, Anna Klimont, Tomasz Wierzbicki, Marek Karczewski, Marek Ruchała, Marcin Rucinski

**Affiliations:** 1 Department of Histology and Embryology, Poznan University of Medical Sciences, 60-781 Poznan, Poland; mszyszka@ump.edu.pl (M.S.); karoljopek01@gmail.com (K.J.); marcinruc@ump.edu.pl (M.R.); 2 Doctoral School, Poznan University of Medical Sciences, 60-812 Poznan, Poland; 3 Department of Endocrinology, Metabolism and Internal Medicine, Poznan University of Medical Sciences, 60-356 Poznan, Poland; hkomar@ump.edu.pl (H.K.); klimont.anna@spsk2.pl (A.K.); mruchala@ump.edu.pl (M.R.); 4 Department of Anatomy and Histology, University of Zielona Góra, Licealna Street 9, 65-417 Zielona Góra, Poland; 5 Department of General, Endocrinological and Gastroenterological Surgery, Poznan University of Medical Sciences, 60-355 Poznan, Poland; tomasz.wierzbicki@ump.edu.pl; 6 Department of General and Transplantation Surgery, Poznan University of Medical Sciences, 60-356 Poznan, Poland; drkarczewski@gmail.com

**Keywords:** humanin, mitochondria, ACC

## Abstract

The discovery of mitochondria-derived peptides (MDPs) has provided a new perspective on mitochondrial function. MDPs encoded by mitochondrial DNA (mtDNA) can act as hormone-like peptides, influencing cell survival and proliferation. Among these peptides, humanin has been identified as a crucial factor for maintaining cell survival and preventing cell death under various conditions. Adrenocortical carcinoma (ACC) is a rare and aggressive malignancy that results from adrenal hormone dysfunction. This study aimed to investigate humanin expression in the adrenal tissue and serum of patients with ACC. For the first time, our study revealed significant reduction in the mRNA expression of humanin in patients with ACC compared to healthy controls. However, no significant changes were observed in the serum humanin levels. Interestingly, we identified a positive correlation between patient age and serum humanin levels and a negative correlation between tumor size and LDL levels. While the impaired expression of humanin in patients with ACC may be attributed to mitochondrial dysfunction, an alternative explanation could be related to diminished mitochondrial copy number. Further investigations are warranted to elucidate the intricate relationship among humanin, mitochondrial function, and ACC pathology.

## 1. Introduction

The traditional function of mitochondria has been revised by the discovery of mitochondrial-derived peptide. This group includes six Small Humanin-Like Peptides (SHLPs 1-6), Humanin and MOTS-c (Mitochondrial Open Reading Frame of the 12S rRNA Type-c) [1,2,3]. Moreover, SHMOOSE and MTALTND4 are newly described mitochondria-encoded microproteins [4,5]. New insights into mitochondrial function include their role as an endocrine protective factor, which may occur as hormone-like peptides [6,7,8]. The first peptide discovered in this group was Humanin (HN). Humanin (HN) is a short, 24-amino acid peptide encoded by a small open reading frame located in the 16S rRNA region of mitochondrial DNA [3]. Humanin was identified over two decades ago (in 2001) and has been recognized for its crucial role in promoting cell survival through neuroprotective and anti-apoptotic activities by suppressing neuronal cell death in Alzheimer’s disease [9]. The primary physiological significance of HN is related to its cytoprotective effect by acting as a cell survival supporter protein, particularly in response to cellular stress [10]. Humanin may also exhibit dynamic translocation to the nucleus (e.g., as a response to cellular stress), proving that microproteins such as humanin may act as nuclear transcriptional factors, influencing gene expression [11,12]. A substantial body of evidence suggests that humanin levels generally decline with age, but its administration has potential benefits in preventing and reversing age-related diseases and conditions [13,14,15,16,17].

Mitochondria are abundant in the adrenal glands and participate in the biosynthesis of aldosterone and cortisol, the primary adrenal hormones [18]. These hormones play an essential role in regulating blood pressure and act in opposition to insulin, thereby participating in the regulation of metabolism. The physiological concentration of adrenal hormones is precisely controlled, and any disturbances in this regulation lead to serious health consequences.

Adrenocortical carcinoma is a rare and aggressive malignancy with an estimated annual incidence of approximately two cases per million people [19,20]. Since 2017, ACC has been classified by the World Health Organization and divided into several subtypes: primary, myxoid, oncocytic, sarcomatoid, and pediatric [21]. The disease most often affects women, and its peak incidence occurs between the ages of 40 and 60 years. Surgery plays a critical role in ACC management and can achieve beneficial outcomes [22]. For patients with inoperable or metastatic ACC, mitotane has been identified as an adjuvant therapy or treatment option. In cases of rapid disease progression among chemotherapy-treated ACC patients, a combination of etoposide, doxorubicin, cisplatin, and mitotane was employed. The clinical manifestations of ACC depend mainly on the hormonal activity of the tumor (overproduction of adrenal hormones) and are characterized by poor prognosis. In addition to ACC, the pathological changes in the adrenal gland also include adrenal cortical adenoma, pheochromocytoma, diffuse adrenal cortical hyperplasia, and very rare neuroblastoma or glioblastoma. Depending on the disease, adrenal tumors might be hormonally inactive, but they may also appear functional, which is related to the excess secretion of adrenal hormones [23,24,25,26,27].

The current literature does not provide information on the contribution of humanin to the progression of adrenocortical carcinoma or other adrenal tumors. This study aimed to fill this gap by investigating the expression of humanin in patients with adrenocortical carcinoma (ACC) at both the gene and protein levels. These findings will be compared with the expression of humanin in normal adrenal tissue, contributing valuable insights into the potential role of humanin in ACC pathology.

## 2. Results

### 2.1. Humanin mRNA Expression Is Significantly Downregulated in Adrenocortical Carcinoma

**Figure 1:**
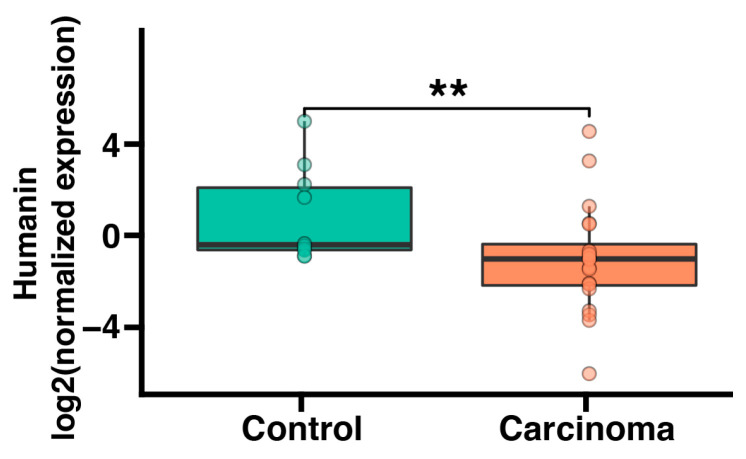
Adrenal expression of humanin mRNA in patients with adrenocortical carcinoma (*n* = 14) compared to normal adrenals (*n* = 6). The bar plot displays the median with interquartile range. Each dot represents humanin expression in individual patients. Statistical differences were determined by using the Mann−Whitney U nonparametric test (** *p* < 0.01).

Initially, we conducted an analysis of humanin gene expression at the mRNA level in both normal human adrenals and adrenocortical carcinoma. Humanin expression was evaluated by quantitative real-time PCR. In both normal adrenal glands and adrenocortical carcinoma, the humanin expression level was relatively high (mean ct value from real-time PCR equaled 28). The expression of humanin in adrenocortical carcinoma was significantly lower than in normal adrenal glands (*p* < 0.01) (Figure 1).

### 2.2. Expression of Humanin at the Protein Level, Determined in Blood Serum, Does Not Differ between the Control Group and ACC

**Figure 2:**
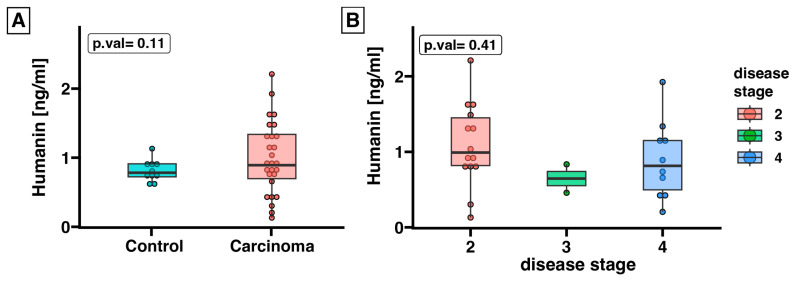
The unchanged level of humanin protein (ng/mL) in serum of ACC patients (*n* = 26) compared to healthy controls (*n* = 10). Humanin levels were measured by using the ELISA test and analyzed based on the presence of the disease (**A**) and disease stage (**B**). Statistical differences were determined by using the Mann−Whitney U test (**A**) or the Kruskal–Wallis test (**B**).

In light of the findings suggesting that humanin could be secreted from cells and detected in the systemic circulation, we assessed humanin protein expression in the serum of ACC patients (*n* = 27) in relation to the control group (*n* = 10). The ELISA assay revealed that the expression of humanin was unchanged in the serum of ACC patients compared to that in healthy controls (*p* = 0.11) (Figure 2A). Furthermore, we conducted an analysis of humanin expression during disease progression and found a lack of significant differences of humanin concentration in patient serum (Figure 2B).

**Figure 3:**
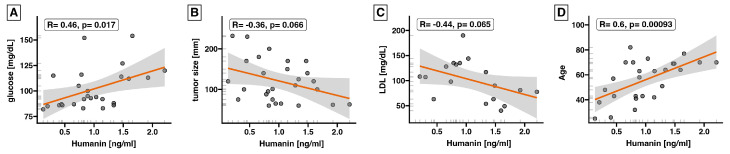
Correlations of humanin protein expression level in serum of ACC patients (*n* = 26) with (**A**) glucose level (R = 0.46, p = 0.017), (**B**) tumor size (R = −0.36, *p* = 0.066), (**C**) LDL serum level (R = −0.44, *p* = 0.065), and (**D**) patients age (R = 0.6, *p* = 0.00093). Each dot represents an individual ACC patient. The orange line represents the regression line.

**Figure 4:**
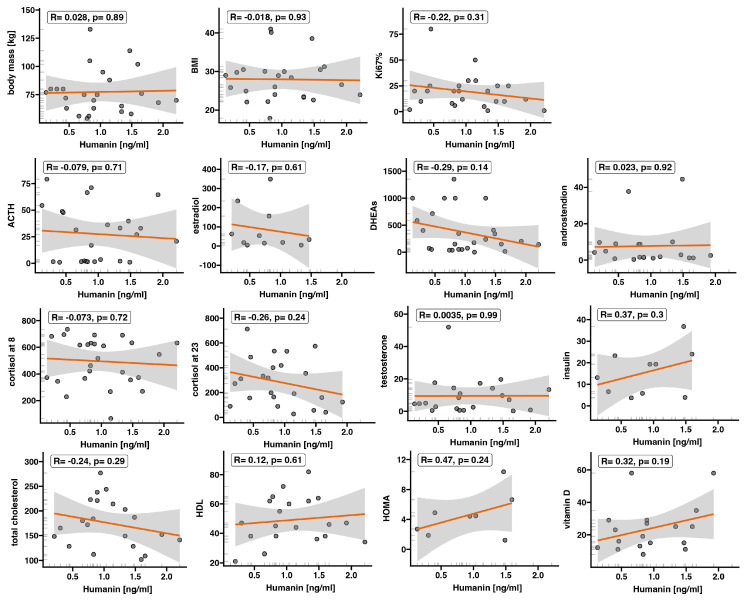
The correlations of humanin protein expression level in serum with clinical characteristics of ACC patients (*n* = 26). Each dot represents an individual ACC patient. The orange line represents the regression line.

Moreover, we explored whether serum humanin levels could be associated with patients’ clinical outcomes (Figure 3 and Figure 4). While serum expression of humanin did not significantly change in patients with ACC, we showed a positive correlation between humanin level and glucose level (*p* = 0.017, R = 0.46) (Figure 3A), as well as with patient age (*p* < 0.001, R = 0.6) (Figure 3D). Additionally, we observed a negative correlation between humanin protein levels and tumor size (*p* = 0.06, R = −0.36) (Figure 3B) and low-density lipoprotein (LDL) levels (*p* = 0.06, R = −0.44) (Figure 3C) in the serum of patients with ACC. However, in both cases the *p*-value was close to statistical significance. We evaluated whether there was a relationship between serum humanin levels and other clinical parameters of the patients (Figure 4); however, we did not find any relationship between humanin expression level and the analyzed parameters, such as patients’ phenotype, hormone level, and biochemical data.

### 2.3. Humanin Expression at the Protein Level in the Adrenal Glands Is Downregulated in the following Groups: Adjacent Normal Adrenal Tissue, Adrenal Cortical Hyperplasia, Adrenal Cortical Adenoma, Adrenal Cortical Adenocarcinoma, and Neuroblastoma and Ganglioneuroma

**Figure 5:**
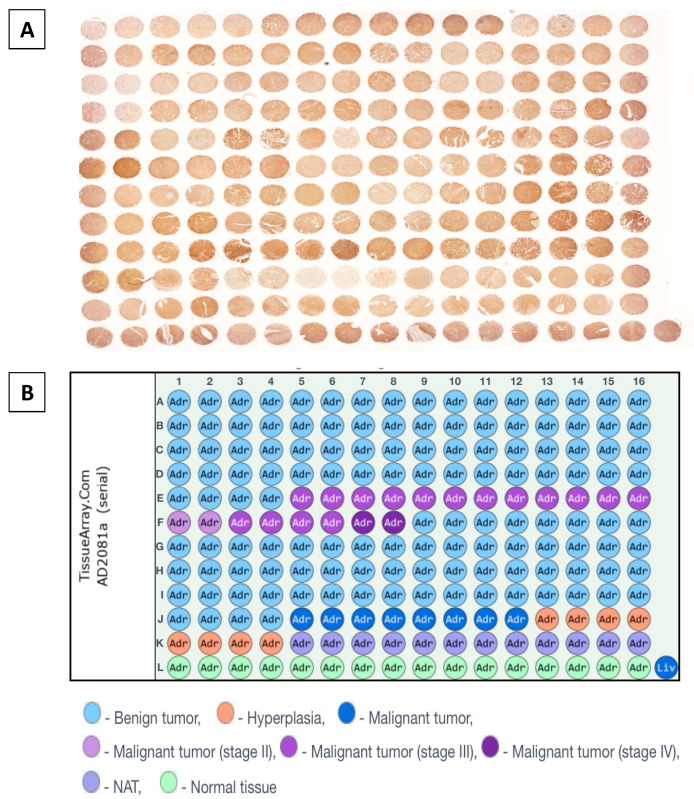
The tissue microarray slide (TMA) was used to analyze the expression of humanin protein in the human adrenal gland disease spectrum, focusing on various types of adrenal cancer. The localization of humanin protein was visualized through immunohistochemical staining (DAB) (**A**). The relevant types of adrenal cancer progression are indicated on the TMA map using distinct colors (**B**).

**Figure 6:**
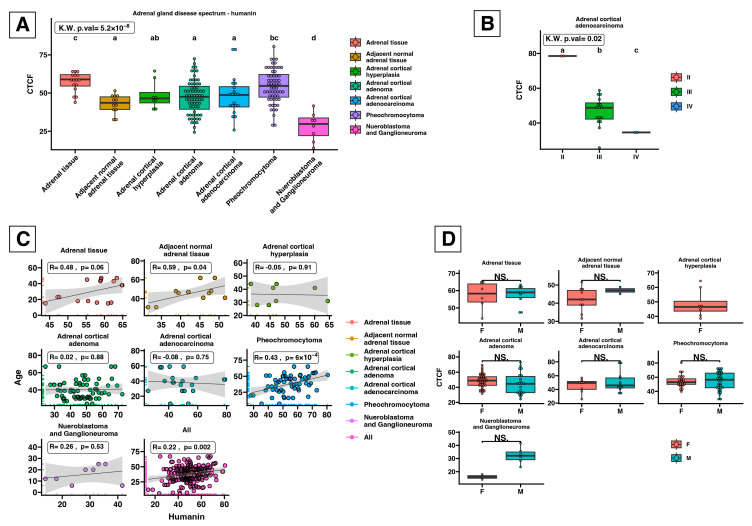
Densitometric analysis of humanin protein expression in the tissue microarray slide of the adrenal gland disease spectrum compared to adrenal tissue (healthy control). The expression of humanin protein was analyzed in tissues from normal adrenal (*n* = 8), adjacent normal (*n* = 6), adrenal cortical hyperplasia (*n* = 4), adrenal cortical adenoma (*n* = 34), adrenal cortical adenocarcinoma (*n* = 10), pheochromocytoma (*n* = 30), neuroblastoma (*n* = 3), and ganglioneuroma (*n* = 1), using duplicate cores per case. The expression of humanin protein is decreased according to ACC progression (**A**,**B**). Correlations of tissue humanin expression level with age of adrenal gland disease spectrum patients (**C**). No significant alterations were observed based on the biological sex of patients with ACC (**D**). The boxplot displays each group with its median and interquartile range (IQR) (**A**,**B**,**D**). Individual patient densitometric data were added to the corresponding boxplots and represented as dots. The Kruskal–Wallis (KW) test was used to compare groups, followed by the Dunn post hoc test. Differences between groups were denoted with letter annotation, where different letters mark statistically significant differences between compared groups.

**Figure 7:**
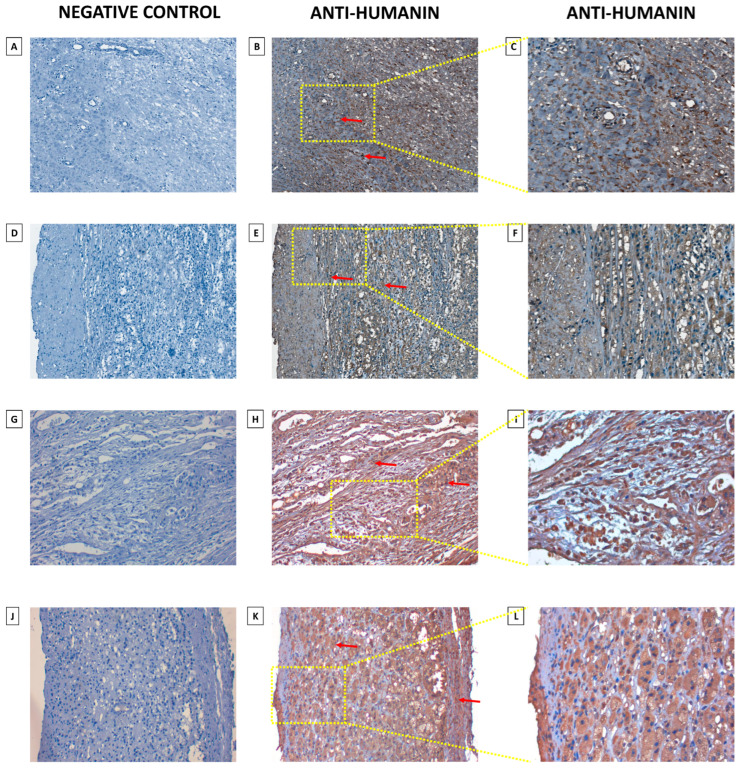
Immunohistochemical analysis of humanin protein of the progression of ACC (stage II (**A**–**F**), stage III (**G**–**I**), stage IV (**J**–**L**)). Brown staining (**B**,**C**,**E**,**F**,**H**,**I**,**K**,**L**) represents humanin protein (red arrows), located typically cellularly with hematoxylin counterstain (nucleus). The negative control of adrenal gland tissue (**A**,**D**,**G**,**J**). The images were captured using 20× (**A**,**B**,**D**,**E**,**G**,**H**,**J**,**K**) and 40× (**C**,**F**,**I**,**L**) objective.

As tissue microarray (TMA) enables the examination of protein expression patterns in various diseased and normal tissues, we investigated alterations in humanin levels within tissues associated with the progression of adrenal cancer (Figure 5). We conducted densitometric analysis of sections that were immunohistochemically stained against humanin (Figure 6), which revealed a decrease in humanin protein expression (*p* = 0.004) and is downregulated with the progression of ACC (*p* = 0.02) (Figure 6B). Furthermore, we indicate that the protein expression of humanin was also diminished in adrenal cortical hyperplasia (*p* = 0.01), adrenal cortical adenoma (*p* < 0.001), and neuroblastoma and ganglioneuroma (*p* < 0.001). Moreover, densitometric analysis revealed that the expression of humanin was strongly positively correlated with the age of patients with pheochromocytoma (*p* = 6 × 10^−4^, R = 0.43), while for ACC and adrenocortical adenoma, we did not indicate any significant correlations. Overall analysis of samples showed a positive correlation with the age of the patients, with elevated expression being more prominent in older individuals (*p* = 0.002, R = 0.22) (Figure 6C). Additionally, we analyzed the potential differences in humanin expression levels based on the biological sex of the patients, but we did not observe any significant changes in this regard (Figure 6D). The localization of humanin in adrenocortical carcinoma tissues mostly presents as cytoplasmic localization of the protein (Figure 7).

**Figure 8:**
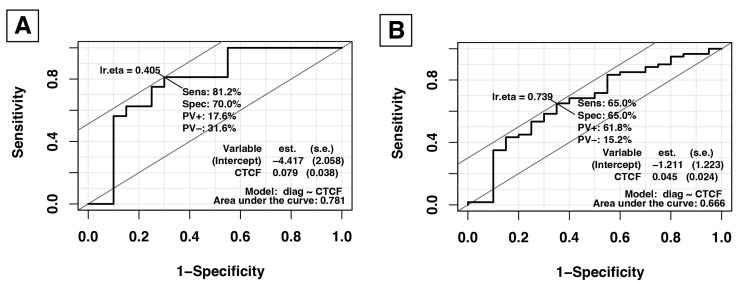
Receiver operating characteristic (ROC) curves for normal vs. cancer (**A**) and pheochromocytoma vs. cancer (**B**) illustrating the potential of HN immunoreactivity as a biomarker in adrenal cancer. The area under curve (AUC) values are shown in graphs.

Moreover, we conducted an analysis of the receiver operating characteristic (ROC) between normal and carcinoma patients to assess the potential of humanin to serve as a diagnostic biomarker for ACC. All relevant values are depicted in Figure 8.

## 3. Discussion

In the present study, we broadly investigated the gene and protein expression of humanin in the tissue and serum of patients with adrenocortical carcinoma. The most conspicuous observation to emerge from the data analysis was that the mRNA expression of humanin was impaired during the ACC conditions compared to healthy controls (*p* = 0.01). While we did not indicate significant changes in the serum level of humanin protein within the pathological stage of ACC, we revealed that glucose level (R = 0.46, *p* = 0.017) and patient age (R = 0.06; *p* = 0.0009) were positively correlated whereas LDL level (R = −0.44; *p* = 0.065) and tumor size (R = −0.36; *p* = 0.06) were negatively correlated with humanin serum level. Moreover, based on TMA slide analysis, we showed that the protein expression of humanin decreased during ACC progression (*p* = 0.02) but did not correlate with the patient’s sex.

To the best of our knowledge, this is the first time that a specific examination of humanin expression in adrenocortical carcinoma has been conducted. Humanin is one of the best-known peptides of mitochondrial origin, but still, its role in cancer is poorly understood. Research suggests that circulating humanin in serum may be a potential diagnostic biomarker for breast cancer or glioblastoma [28,29]. Humanin is overexpressed in biopsies from patients with gastric [30] and bladder cancers [31] when compared to non-neoplastic surrounding tissues.

Warburg’s effect, characterized by increased glycolysis, decreased mitochondrial oxidative phosphorylation, and lactate production in cancer cells, promotes tumor proliferation [32,33,34,35]. In contrast to this theory, mitochondrial function is crucial for cancer cell survival, whereas elimination of mtDNA from cancer cells slows down their growth and compromises tumorigenesis [36]. The concentration of humanin released by mitochondria is regulated to achieve homeostasis. Properly functioning mitochondria are responsible for limiting the harmful effects of cellular stress, which, according to the definition of hormesis, depends on stress intensity [37,38]. Depending on the intensity and frequency of stressful conditions, mitokines can either strengthen the body (in the case of mild stress) or affect metabolic stress in the event of excessive secretion. The theory by Conte et al. assumes that, as a result of an adaptive mechanism, the role of mitokines changes from protective to harmful as we age. Therefore, an increase in humanin concentration attempts to counteract the detrimental effects of mitochondrial degradation. Our findings seem to demonstrate that the reduced humanin mRNA level in the adrenals of ACC patients might contribute to the altered mitochondrial function and increased oxidative stress observed in ACC.

Human aging, usually consistent with mitochondrial dysfunction caused by increased mitophagy and mitokine secretion, initiates crucial stress response mechanisms that might have a wholesome effect [39]. Despite a growing body of evidence indicating that humanin level (in rodents and humans) declines during aging, contributing to age-related diseases, our current study does not support previous research in this area [40,41]. Contrary to what was previously thought, we found that humanin serum levels increase with age, which is consistent with Conte et al. [42]. Interestingly, another study indicates that descendants with exceptionally long-lived family histories, particularly the offspring of centenarians, revealed enhanced circulating humanin levels. These findings tempt us to consider circulating humanin levels as a determinant of increased life expectancy [14,43]. Lytvyn et al. suggested that humanin levels may be sex-specific, proving that humanin concentrations are higher in the serum of healthy women [44]. However, loss of humanin has been observed during diabetes mellitus (type 1) [44]. It is attributed to enhanced oxidative stress in women with diabetes mellitus, possibly caused by free radicals or a deficiency in the antioxidant defense system [45,46]. All of these factors can contribute to mitochondrial dysfunction and decrease humanin levels. This context is supported by considerable research indicating that mitochondrial dysfunction is crucial for cancer progression [47,48]. As a stress response, mitochondria can generate pathways and molecules that can act as influential regulators of retrograde signaling in the progression of cancer [36,49,50]. Simultaneously, mitochondria undergo metabolic changes to promote cancer cell proliferation and formation, contrary to Warburg’s hypothesis [32,33]. The copy number of mitochondrial DNA (mtDNA) also changes during cancer development. Therefore, in the case of colorectal cancer [51], endometrial adenocarcinoma [52], and glioma [53], an increase in mtDNA was proven, while in the case of hepatocellular carcinoma [54] and breast cancer [55], a decrease in mtDNA copy number was observed. Therefore, a reduced number of mitochondria may hinder an organism’s ability to prevent the effects of the disease, leading to its progression.

Furthermore, gonadal steroids exert an inhibitory effect on humanin expression suggesting that gonadal steroids are involved in the regulation of humanin expression [56]. Nevertheless, the lack of sexual dimorphism in humanin plasma secretion observed in our study is consistent with previous findings [42]. Interestingly, supplementation of humanin in a freezing-thawing medium facilitates sperm motility and viability [57]. Humanin has been shown to improve sperm condition by reducing ROS, caspase-3 activity, and mitochondrial membrane integrity [57]. Moreover, humanin may promote steroidogenesis and survival of Leydig cells of rats [58]. These reports suggest that humanin may be a potential protective factor in maintaining spermatogenesis and steroidogenesis homeostatis.

Herein, we indicate that circulating humanin levels are positively correlated with glucose concentration in the serum of patients with ACC. Recent studies of insulin resistance and β-cell dysfunction have suggested the occurrence of mitochondrial dysfunction and diminished mtDNA copy number [1,59,60,61,62,63,64]. Moreover, humanin is a novel cytoprotective hormone that inhibits *Bax* and cytochrome C release in the cell apoptosis pathway and can protect pancreatic β-cells. Humanin treatment improves glucose tolerance, reduces visceral fat, and increases glucose-stimulated insulin release [7,65]. Moreover, it has been reported that plasma humanin levels are decreased in people with type 2 diabetes, potentially making it a therapeutic option against this condition [62,66]. Although our research does not confirm previous literature data, we assume that the positive correlation between the level of humanin and glucose in patients with ACC manifests an adaptation mechanism related to ongoing disease. Further investigation of humanin molecular action is crucial to fully understand the role of MDPs in ACC.

We are aware that our research has some limitations. The first is the small number of patients with ACC enrolled in this study; however, the classification of ACC as a rare disease provides a unique and homogeneous group in the context of clinical characteristics. Second, the sample collection method allowed us to analyze gene expression only, thus excluding the possibility of in-depth analyses of protein expression. The third thing that needs to be mentioned is that circulating humanin in serum is released not only by adrenals but also from all tissues. However, further research is necessary to elucidate the role of humanin in adreno-cortical carcinoma diagnosis and progression.

As far as we know, this is the first report on the expression of humanin in the adrenal gland tissues of patients with ACC. The three principal conclusions of this study were (*i*) the expression of humanin is inhibited in the adrenals of patients with ACC, (*ii*) the expression of humanin protein decreases during ACC progression, and (*iii*) the plasma level of humanin increases with ACC patients’ age. There are several possible explanations for these results. On the one hand, the decreased level of humanin during ACC may suggest an impaired molecular mechanism of mitochondrial functioning and activity. However, on the other hand, it may be caused by diminished mitochondrial copy number. Taken together, we hypothesize that decreased expression of humanin, both at the gene and protein levels, could be a progressive factor in poor ACC prognosis. Further studies are needed to elucidate the underlying mechanisms and potential clinical applications of humanin in ACC.

## 4. Materials and Methods

### 4.1. Patients’ Characteristics

In this research, 26 patients were recruited for the study with necessary clinical data over the years from 2011 to 2023. All 26 patients’ serum was collected, aliquoted and stored at -80°C for further ELISA analysis (*n* = 26). From 14 patients who had undergone adrenalectomy because of suspected adrenocortical carcinoma (ACC) we collected pathologically changed adrenal specimens (~0.5 cm3) tissue and preserved it in RNAlater (#R0901, Sigma, St. Louis, MO, USA) for mRNA analysis (*n* = 14). The control group (*n* =6) consisted of tissue fragments and serum from kidney donors (*n* = 6) and whole blood of healthy donors (*n* = 10). The Ethics Committee (Institutional Review Board) of the Poznan University of Sciences (reference number of the decision: 31/22) approved the study, and all subjects provided informed written consent. The study protocol conformed to the ethical guidelines of the Declaration of Helsinki. The patients’ clinical characteristics were described earlier [67].

### 4.2. RNA Isolation and qPCR Performing

The RNA was extracted from tissues using TRIzol (Invitrogen, Waltham, MA, USA) as per the manufacturer’s instructions. Next, to obtain small RNA (<200 nucleotides) fragments, we used dedicated columns from the miRNeasy Tissue/Cells Advanced Micro Kit (#217684, Qiagen, Hilden, Germany). The RNA stock concentration was adjusted to 1 μg/μL based on the absorbance measurement at 260 nm in water, with an assumption that an absorbance of 1 corresponds to RNA at 40 μg/mL. Quality control was performed on all RNA samples, including checking for purity (A260:A280) > 1.8. Next, the 500 μg/mL of RNA were used for the reversed transcription reaction to obtain cDNA. The reverse transcription reaction was based on the TaqMan™ MicroRNA Reverse Transcription Kit (#4366596, ThermoFisher, Carlsbad, CA, USA) in accordance with the manufacturer’s instructions. The obtained cDNA were stored at −20 °C. Furthermore, the quantitative Real-time PCR reaction was performed in duplicates on the CFX96 Touch (Bio-Rad, Contra Costa County, CA, USA). For the qRT-PCR reaction, TaqMan™ Fast Advanced Master Mix (#4444557) with humanin (4440886) and RNU48 (4427975, assay ID: 001006) assays were used. The humanin assay was predesigned using the Custom TaqMan^®^ Small RNA Assay Design Tool against the humanin mtDNA sequence and confirmed by a ThermoFisher specialist. The expression of humanin mRNA was normalized to the genome RNU48 using the ΔΔCt method.

### 4.3. ELISA

The enzyme-linked immunosorbent assay (ELISA) measured humanin protein serum concentration (SET833Hu, Cloud-Clone Corp., Katy, TX, USA). The aliquoted serum of adrenocortical carcinoma patients and healthy donors was stored at −80 °C before undergoing analysis. We used the ELISA kit following the manufacturer’s instructions. All measurements were performed in duplicates.

### 4.4. Immunohistochemical Analysis

The localization of humanin protein was analyzed through immunohistochemical examinations of tissue microarray (TMA). TMA slides obtained from US Biomax Inc. (AD2081a; Rockville, MD, USA) represent human normal and adrenal gland disease spectrum (adrenal gland cancer progression) cases. The procedure of deparaffinization and staining was described earlier [67]. In brief, the TMA slide was deparaffinized in xylene and then incubated twice in 100% and once in 96%, 80%, and 70% of ethanol for 1 min at RT. Next, to enhance the staining intensity of antibodies, we performed antigens and epitopes unmasking in Citrate buffer solution (pH 6.1; H-3300-250; Vector Laboratories, Inc.; Newark, CA, USA) in a microwave for 15 min and cooled to the RT. Then we performed the staining procedure following the manufacturer’s protocol of ImmPRESS^®^ HRP Universal (Horse Anti-Mouse/Rabbit IgG) PLUS Polymer Kit (MP-7800; Vector Laboratories, Inc.; Newark, CA, USA). The TMA slides were incubated with an anti-Humanin antibody with 1:250 dilution (#25886-1AP; Proteintech Group, Inc.; Rosemont, IL, USA) at 4 °C overnight. Moreover, we performed counterstaining with Mayer’s hematoxylin (#S330930-2, DAKO, CA, USA), dehydrated, and mounted slide. The TMA slide was digitalized with a Grundium Ocus^®^20 Microscope Scanner (Tampere, Finland). The analysis of documented IHC staining was accomplished by using CaseViewer 2.3 (64-bit version) for Windows (3D Histech Ltd., Budapest, Hungary).

### 4.5. Densitometric Analysis of Tissue Microarray Slide and Statistical Analysis

Densitometric analysis of humanin protein expression involved normal adrenal gland, adenomas, and adrenocortical carcinomas groups. In the first step, we removed the blue-violet color (hematoxyline staining) from the TMA image. Next, the brown dye (corresponding to the IHC reaction) was converted to grayscale using the color inversion method in Adobe Photoshop ver. 21.1.0 (Adobe Inc., San Jose, CA, USA). Then, the densitometric analysis of the image in TIFF format was performed by using ImageJ software (version 1.5q, Wayne Rasband, National Institutes of Health, Bethesda, MD, USA), following The Open Lab Book protocol adapted to TMA format [68].

The obtained pixel intensity results of each tissue array core were used for statistical analysis in the R programming language (version 4.1.2; R Core Team 2021) supported by the “ggplot2” library for results visualization. The results of our analysis were presented as boxplots, with median and interquartile range (IQR), where each dot represents an individual patient. The Kruskal–Wallis test, followed by the Dunn post hoc test, was used to compare results obtained from two groups. The differences between groups were estimated using the letter annotation, where different letters indicate significant differences (*p* < 0.05). The *p*-value of the post-hoc test for each pairwise comparison was also shown. Pearson correlation with an r-coefficient value and linear regression were generated using a “ggplot2” library and were used to examine the associations between serum humanin concentration and clinical data outcomes.

## Data Availability

All of the data discussed in this work, if not already included in the manuscript, are available from the corresponding author on reasonable request.
